# Increase in Testosterone Levels and Improvement of Clinical Symptoms in Eugonadic men With a Prolactin-secreting Adenoma

**DOI:** 10.1210/jendso/bvae135

**Published:** 2024-07-16

**Authors:** Lea Carlier, Philippe Chanson, Laure Cazabat, Sylvie Daclin, Sylvie Salenave, Mirella Hage, Séverine Trabado, Jacques Young, Luigi Maione

**Affiliations:** Department of Endocrinology and Reproductive Diseases, Université Paris-Saclay, Inserm UMRS1185, Physiologie et Physiopathologie Endocriniennes, Assistance Publique-Hôpitaux de Paris, Hôpital Bicêtre, Service d'Endocrinologie et des Maladies de la Reproduction, 94275 Le Kremlin-Bicêtre, France; Department of Endocrinology, Hôpital Ambroise Paré, Assistance Publique-Hôpitaux de Paris, 92100 Boulogne Billancourt, France; UE4340, Université de Versailles Saint-Quentin-en-Yvelines Montigny-le-Bretonneux, 78000 Versailles, France; Department of Endocrinology and Reproductive Diseases, Université Paris-Saclay, Inserm UMRS1185, Physiologie et Physiopathologie Endocriniennes, Assistance Publique-Hôpitaux de Paris, Hôpital Bicêtre, Service d'Endocrinologie et des Maladies de la Reproduction, 94275 Le Kremlin-Bicêtre, France; Department of Endocrinology, Hôpital Ambroise Paré, Assistance Publique-Hôpitaux de Paris, 92100 Boulogne Billancourt, France; UE4340, Université de Versailles Saint-Quentin-en-Yvelines Montigny-le-Bretonneux, 78000 Versailles, France; Department of Endocrinology and Reproductive Diseases, Université Paris-Saclay, Inserm UMRS1185, Physiologie et Physiopathologie Endocriniennes, Assistance Publique-Hôpitaux de Paris, Hôpital Bicêtre, Service d'Endocrinologie et des Maladies de la Reproduction, 94275 Le Kremlin-Bicêtre, France; Department of Endocrinology and Reproductive Diseases, Université Paris-Saclay, Inserm UMRS1185, Physiologie et Physiopathologie Endocriniennes, Assistance Publique-Hôpitaux de Paris, Hôpital Bicêtre, Service d'Endocrinologie et des Maladies de la Reproduction, 94275 Le Kremlin-Bicêtre, France; Department of Endocrinology, Hôpital Ambroise Paré, Assistance Publique-Hôpitaux de Paris, 92100 Boulogne Billancourt, France; UE4340, Université de Versailles Saint-Quentin-en-Yvelines Montigny-le-Bretonneux, 78000 Versailles, France; Department of Molecular Genetics, Pharmacogenetics and Hormonology, Université Paris-Saclay, Inserm UMRS1185, Physiologie et Physiopathologie Endocriniennes, Assistance Publique-Hôpitaux de Paris, Hôpital Bicêtre, Service de Génétique Moléculaire, Pharmacogénétique et Hormonologie, 94275 Le Kremlin-Bicêtre, France; Department of Endocrinology and Reproductive Diseases, Université Paris-Saclay, Inserm UMRS1185, Physiologie et Physiopathologie Endocriniennes, Assistance Publique-Hôpitaux de Paris, Hôpital Bicêtre, Service d'Endocrinologie et des Maladies de la Reproduction, 94275 Le Kremlin-Bicêtre, France; Department of Endocrinology and Reproductive Diseases, Université Paris-Saclay, Inserm UMRS1185, Physiologie et Physiopathologie Endocriniennes, Assistance Publique-Hôpitaux de Paris, Hôpital Bicêtre, Service d'Endocrinologie et des Maladies de la Reproduction, 94275 Le Kremlin-Bicêtre, France

**Keywords:** acquired hypogonadism, testosterone, puberty, pituitary tumor, infertility

## Abstract

**Objective:**

Testosterone concentrations, albeit rarely, may be in the normal range (>3.0 ng/mL) in men with a prolactin-secreting pituitary adenoma (PSPA-nt). The evolution of total, bioavailable testosterone, gonadotropin levels, and that of graded symptoms of testosterone deficiency (TD) are uncertain in these patients.

**Design:**

Retrospective case-control longitudinal study at a tertiary referral center.

**Methods:**

From 287 men, we selected 25 PSPA-nt men undergoing prolactin normalization (<20.0 ng/mL) during the follow-up. Graded symptoms of TD were investigated by structured interviews. Biochemical changes and TD symptoms were compared to those of a matched cohort of 61 men with pituitary neoplasms and normal testosterone levels (PA-nt).

**Results:**

Baseline testosterone levels were similar between PSPA-nt and PA-nt subjects. The prevalence of specific and suggestive symptoms of TD was higher in PSPA-nt (20% and 68%) than in PAnt (3.3 and 29.5%; *P* = .02 and *P* = .0015, respectively). At the follow-up, total and bioavailable testosterone levels increased in PSPA-nt but not in PA-nt patients (Δ change: 1.28 ± 2.1 vs0.03 ± 1.5 ng/mL, + 0.33 ± 0.55 vs-0.26 ± 0.60 ng/mL; *P* = .0028 and *P* = .0088, respectively). LH and FSH levels also increased in PSPA-nt men (*P* < .05). Specific and suggestive, but not nonspecific symptoms of TD, improved only in PSPA-nt men (*P* < .05 for both). Baseline testosterone and LH were the strongest predictors of testosterone improvement in PSPA-nt patients.

**Conclusion:**

Despite having normal testosterone levels at baseline, patients with PSPA-nt experience a relief of TD symptoms and an improvement of their pituitary-gonadal axis function following prolactin normalization, especially when baseline TT and LH levels are in the low-normal range.

Prolactin excess represents a leading cause of acquired hypogonadotropic hypogonadism in men [1-5] because of a multimodal inhibitory effect of prolactin on the hypothalamus-pituitary-gonadal (HPG) axis [6, 7]. However, the extent of gonadotropic deficiency in men is wide and variable, ranging from very low to near-normal testosterone levels [8]. Functional inhibition of the HPG axis is demonstrated by the recovery of testosterone levels and by the improvement in signs and symptoms of testosterone deficiency (TD) in nearly half of hypogonadal patients with prolactin-secreting pituitary adenoma (PSPA) receiving effective treatments to reduce prolactin levels [5]. Nonetheless, a small fraction of patients with PSPA may have biochemically normal testosterone levels (>2.7 or 3.0 ng/mL) despite having very high peripheral prolactin concentrations [9]. The prevalence and the evolution of signs and symptoms of TD, as well as the evolution of hormone levels, are uncertain in these patients [9, 10]. We conducted a longitudinal hormonal and clinical evaluation in men with PSPA and repeatedly normal testosterone levels at baseline (PSPA-nt) to explore whether an effective treatment with dopamine agonists could still be associated with a significant increase in testosterone levels. We also evaluated the presence of specific, suggestive, and nonspecific signs and symptoms of TD to assess the clinical significance of the observed changes in these patients. An age-, body mass index (BMI)-, and follow-up duration-matched cohort of men with a nonprolactin-secreting pituitary lesion and repeatedly normal testosterone levels (PA-nt) was used as main comparator to assess testosterone and clinical symptoms changes over a follow-up of the same duration.

## Patients and Methods

### Patients

This is a retrospective longitudinal study conducted at a tertiary referral center (Reference Center for Pituitary Rare Diseases CRMR-Hypo, Bicetre Hospital, France, affiliated with the ENDO-ERN network). From a large cohort of patients harboring PSPA, we included those having, on 2 or more occasions, serum total testosterone concentrations ≥3.0 ng/mL (≥10.4 nmol/L, PSPA-nt), according to generally recommended clinical practices [11, 12]. We excluded patients with no longitudinal evaluation. Importantly, because the threshold at which prolactin produces a functional inhibition on the hypothalamus-pituitary-gonadal (HPG) axis is uncertain, we included only PSPA-nt patients having attained strictly normal prolactin levels (<20 ng/mL) at the last evaluation. A flowchart illustrating the number of patients included and excluded from the study is provided in Supplementary Fig. S1 [13].

To take into account the biologic variability of testosterone assay and the clinical significance of this marker's evolution, we decided to include an age-, BMI-, and follow-up duration-matched cohort of individuals harboring a nonprolactin-secreting pituitary adenoma (PA-nt). Etiologies of PA-nt patients are provided in Supplementary Table S1 [13]. For patients with PSPA to be included, PA-nt patients had to have morning serum total testosterone concentrations repeatedly ≥3.0 ng/mL (≥10.4 nmol/L). In addition, we excluded patients with mildly elevated prolactin levels attributed to a pituitary stalk compression and those with untreated anterior pituitary deficiencies. We did not include patients having had pituitary surgery or radiotherapy occurring between the recruitment period and the last visit. We finally excluded patients undergoing pharmacologic castration or testosterone-lowering agents prescribed for other occurring diseases.

The main demographic, clinical, and biochemical characteristics of patients with PSPA-nt and PA-nt included in this study are shown in Table 1.

**Table 1.** Main demographic, clinical, and hormonal characteristics of men harboring a PSPA (PSPA-nt) and a nonprolactin-secreting pituitary tumor (PA-nt) with total testosterone levels in the normal range, at baseline

	PSPA-nt (n = 25)	PA-nt (n = 61)	*P*
Age at inclusion (y)	44.3 ± 20.4	48.6 ± 11.6	.10
Weight (kg)	82.6 ± 17.3	89.2 ± 17.3	.11
BMI (kg/m^2^)	26.2 ± 4.2	27.2 ± 4.1	.40
Major pituitary tumor axis on MRI (mm)	18.7 ± 11.5	12.4 ± 8.4	.05
Mean testicular volume (mL)	23.7 ± 9.5	20.3 ± 5.5	.62
**Hormone levels**			
Prolactin (ng/mL)	1502.8 ± 2666	10.9 ± 7.5	<.0001
Total testosterone (ng/mL)	4.6 ± 1.3	4.7 ± 1.3	.80
Total estradiol (pg/mL)	16.8 ± 8.3	20.2 ± 7.3	.55
Bioavailable testosterone (ng/mL)	1.29 ± 0.51	1.45 ± 0.65	.81
SHBG (nmol/L)	35.9 ± 12.5	32.6 ± 16.1	.57
FSH (IU/L)	3.6 ± 2.6	7.1 ± 4.5	<.0001
LH (IU/L)	3.0 ± 1.6	3.5 ± 1.8	.19
AMH (pmol/L)	40.7 ± 33.4	48.8 ± 41.2	.30
Inhibin B (pg/mL)	191.9 ± 60.7	172.6 ± 78.4	.40
INSL3 (pg/mL)	687.3 ± 167.7	925.9 ± 421.3	.45
FT4 (pmol/L)	13.5 ± 1.9	15.3 ± 4.0	.10
Cortisol (ng/mL)	158.5 ± 89.7	134.5 ± 42.8	.70
IGF-I (ng/mL)	211.1 ± 226	288.7 ± 219.7	.06

Abbreviations: BMI, body mass index; MRI, magnetic resonance imaging; PA-nt, patients with a nonprolactin-secreting pituitary neoplasm and testosterone levels in the normal range (see also Patients and Methods section); PSPA, prolactin-secreting pituitary adenoma; PSPA-nt, patients with a prolactin-secreting pituitary adenoma and testosterone levels in the normal range.

This study was conducted in accordance with the Declaration of Helsinki. All patients were informed by a notice explaining the type of the study, its scopes and aims, and the handling of sensitive confidential data with information about the right of opposition (according to the French Regulations on data protection, from the Commission Nationale de l'Informatique et des Libertés, see also https://www.cnil.fr/fr/les-bases-legales/consentement). The study protocol has been carefully scrutinized and approved by the local ethics committee (Paris Saclay institutional review board POLETHIS, with annotation no. CER-Paris-Saclay-2023-025, issued on April 20, 2023). In addition, information notices have been widely distributed and posted in public spaces of the Reference Center for Pituitary Rare Diseases CRMR-Hypo.

### Assessment of Signs and Symptoms of TD

Graded signs and symptoms of TD were systematically assessed in PSPA-nt and PA-nt patients at the inclusion and at the last visit. According to the Endocrine Society guidelines, signs and symptoms of TD were graded as specific, suggestive, and nonspecific in each patient [11, 14, 15]. In patients with PSPA-nt, the presence of specific, suggestive, and nonspecific symptoms of TD were assessed again at the time of prolactin normalization. Pubertal development was graded by Tanner staging, and testicular volume was assessed by means of the Prader orchidometer by experienced endocrinologists (P.C., J.Y., and L.M.).

### Biochemical Assays

Serum prolactin was measured by an automated competitive 2-step immunofluorescent assay (Brahms Kryptor GmbH, Hennigsdorf, Germany). This method is devoid of high-dose related hook effect [16]. The intra- and interassays coefficients of variation (CV) were respectively 8.5% and 13.0% for a prolactin concentration of 1.8 ng/mL and 2.4% and 4.3% for a prolactin concentration of 10.3 ng/mL. The lower detection limit was 0.2 ng/mL.

Serum gonadotropins, FSH and LH, were measured by a chemiluminescent immunometric assay (ADVIA Centaur, Siemens, Deerfield, IL, USA). The intra- and inter-assay CVs were 2.9% and 2.7%, respectively, for FSH (at 6.9 IU/L) and 2.3% and 1.5% for LH (at 4.2 IU/L) (expressed relative IS 94/632 and IS 80/552, respectively). The detection limits were 0.3 IU/L for FSH and 0.07 UI/L for LH.

Total testosterone in sera was measured by a sensitive radioimmunoassay using Orion Diagnostica (Spectria, Espoo, Finland) [17]. The lower limit of detection was 0.02 ng/mL (0.06 nmol/L). The intra- and inter-assay CVs were 3.8% and 4.8% for a control serum measuring 2.6 ng/mL (9.1 nmol/L) and 7.5% and 7.0% for 0.35 ng/mL (1.2 nmol/L), respectively [17].

Sex hormone-binding globulin was measured by a solid-phase chemiluminescent immunometric assay (Immulite; Siemens Healthcare Diagnostic Products, Llanberis, United Kingdom) with a detection limit of 0.02 nmol/L. The intra- and interassay CVs were 3.2% and 4.6% for a SHBG concentration of 56.4 nmol/L, as previously reported [2].

Bioavailable testosterone (BT) concentrations were calculated with the algorithm based on the equations of Vermeulen, using the measured total testosterone and SHBG concentrations, an assumed constant for the albumin concentration (43 g/L), and affinity constants of SHBG and albumin for testosterone [18].

Circulating levels of inhibin B, AMH, and INSL3, as well as those of cortisol, IGF-I, and thyroid hormones, were measured as previously reported [2].

### Statistical Analyses

Results are reported as individual values or as means and SDs in the text, figures, and tables. For PSPA-nt patients, the power population was calculated at n = 23 for a before-after study (paired *t*-test) including a 2-tailed alpha at 0.05 and a beta at 0.2 (https://sample-size.net/), after analyzing individual data of 9 patients from a previous study showing a testosterone delta-change (after-before) of 2.3 ± 1.6 ng/mL [9].

In individuals having 4 or more repeated determinations of total testosterone concentrations, variabilities were calculated in each patient as CVs, according to the following formula: CV = [(total testosterone SD)n/(total testosterone average)n]%, where n indicates the number of available determinations, as previously reported [19].

Continuous variables, like hormone levels, age, and BMI, were submitted to the Anderson‒Darling normality test; because they were assessed as nonnormally distributed, nonparametric tests were conducted for all statistical analyses. Wilcoxon matched-pairs signed-rank test was used to compare evolutive changes (before-after) of hormone levels in the same patients (PSPA-nt and PA-nt groups). Mann-Whitney *U* test was used to conduct unpaired comparisons on continuous variables. Fisher exact test was used to compare differences in categorical values between PSPA-nt and PA-nt. Spearman rank procedures were carried out to define correlations between variables. Statistical significance was set at *P* < 0.05.

Graphic representations were generated using GraphPad Prism software, version 6.0 (GraphPad Prism package, La Jolla, CA).

## Results

### Baseline Characteristics of PSPA-nt and PA-nt Patients

From 278 men harboring a PSPA followed at our tertiary referral center between 2011 and 2022, 25 (8.7%) met all inclusion criteria (repeatedly normal serum total testosterone concentrations at baseline, prolactin normalization at the last visit, available testosterone measurements at the time of prolactin normalization). The flowchart illustrating patient inclusion is provided in Supplementary Fig. S1 [13].

From 619 men with a pituitary lesion other than a PSPA actively followed in our Institute, we selected 154 patients (PA-nt) with the same follow-up duration and follow-up period, and with repeatedly normal testosterone levels at baseline. These patients had neither pituitary surgery nor radiotherapy within the follow-up period. They had normal prolactin levels and normal or correctly supplemented pituitary function. From these, we included 61 patients with available testosterone measurement matching PSPA-nt men on age and BMI. The flowchart of PA-nt patient inclusion is illustrated in the Supplementary Fig. S1 [13]. The etiologies of pituitary lesion subtypes in PA-nt patients are reported in the Supplementary Table S1 [13].

No patient from the PSPA-nt and PA-nt groups received androgen substitutes, gonadotropin therapy, or pharmacologic castration over the follow-up duration.

At baseline, PSPA-nt patients tended to have larger adenoma major axis (18.7 ± 11.5 vs 12.4 ± 8.4 mm, *P* = .05, Table 1). As for inclusion criteria, age, weight, and BMI were similar between PSPA-nt and PA-nt patients (Table 1). Baseline total testosterone levels were not different between PSPA-nt and PA-nt subjects (4.6 ± 1.3 vs 4.7 ± 1.3 ng/mL, *P* = .8), as well as total estradiol, BT, SHBG, and LH levels, testicular peptides, and other anterior pituitary hormone levels except prolactin (Table 1). Overall, mean testicular volumes did not differ between PSPA-nt and PA-nt patients (*P* = .62; see Table 1). The mean follow-up duration between the first and the last visit was 26.5 ± 32.9 vs 23.5 ± 14.7 months in PSPA-nt and PA-nt patients, respectively (*P* = .15).

### Evolution of Hormone Levels in PSPA-nt and PA-nt Patients

**Figure 1.**
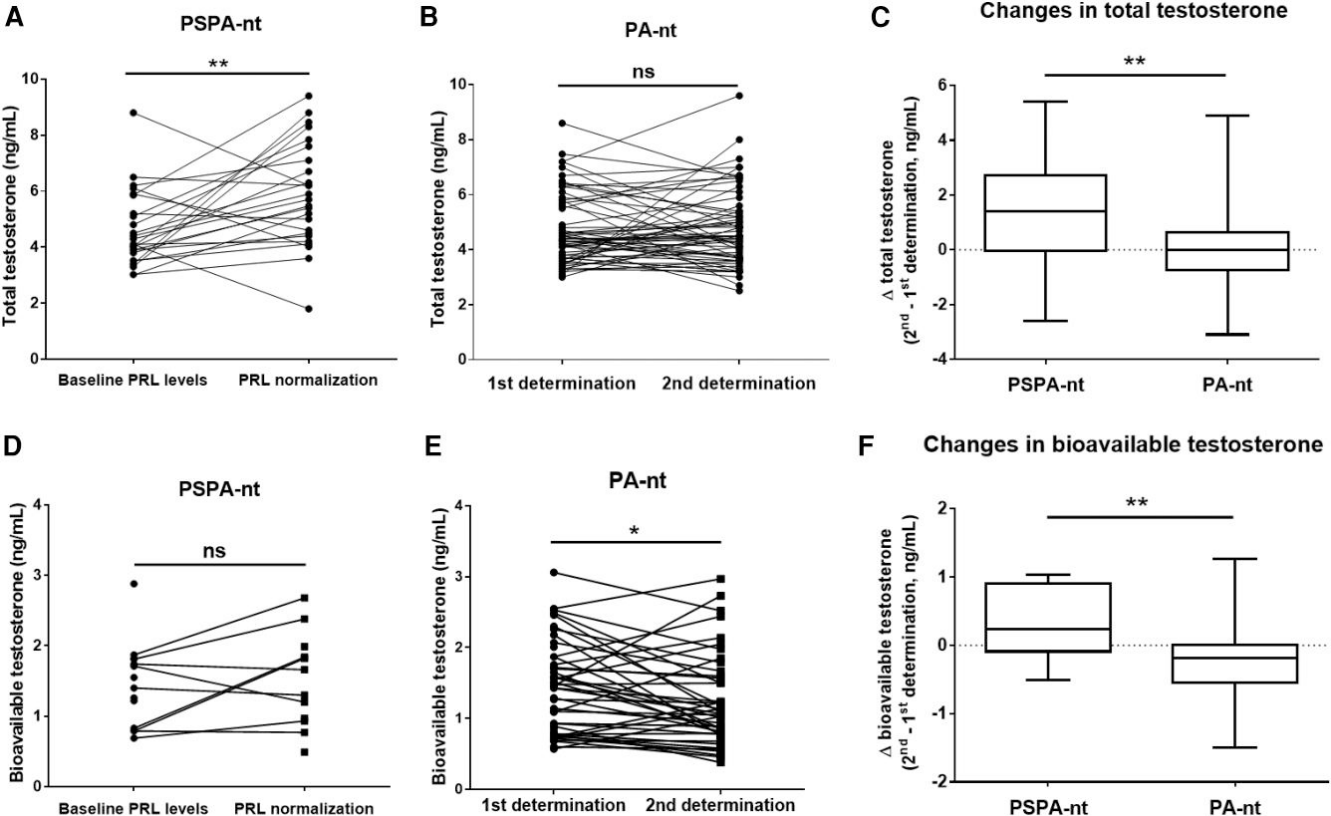
Evolution of serum total and bioavailable testosterone levels in PSPA-nt and PA-nt patients. (A) Total testosterone levels were measured before and after prolactin normalization in patients harboring a prolactin-secreting adenoma and testosterone levels in the normal range (PSPA-nt, n = 25) and at (B) 2 different determinations over a similar follow-up duration (26.5 ± 32.9 vs 23.5 ± 14.7 months, *P* = ns) in patients with a nonprolactin-secreting pituitary neoplasm (PA-nt, n = 61). (C) Changes in total testosterone levels (Δ testosterone) between the last and the first determination in the 2 groups. (D) Bioavailable testosterone levels were calculated before and after prolactin normalization in PSPA-nt patients (n = 9 for paired tests) and at (E) 2 different determinations in PA-nt patients (n = 42 for paired tests) after a similar follow-up duration. (F) Changes in bioavailable testosterone levels (Δ) between the last and the first determination in the 2 groups of patients. ns, not significant. *P < .05; **P < .01.

In PSPA-nt patients, total testosterone levels increased from 4.6 ± 1.3 to 5.9 ± 1.8 ng/mL at the time of prolactin normalization under 0.7 ± 0.6 mg weekly of cabergoline (*P* = .0066; Fig. 1A). Conversely, in PA-nt subjects, total testosterone levels did not significantly change between the first and the second determination over a similar follow-up duration (4.7 ± 1.3 and 4.7 ± 1.4 ng/mL; *P* > .9; Fig. 1B). Consequently, changes in total testosterone levels between the second and the baseline determination (Δ testosterone) were higher in PSPA-nt than in PA-nt patients (+1.28 ± 2.1 vs + 0.03 ± 1.5 ng/mL; *P* = .0028; Fig. 1C).

BT levels were calculated in a subgroup of PSPA-nt and PA-nt patients. BT levels did not significantly change in PSPA-nt (n = 9; from 1.29 ± 0.51 to 1.62 ± 0.64 ng/mL; *P* = .2; Fig. 1D), but slightly decreased in PA-nt patients between the first and the second determination (n = 42; from 1.45 ± 0.65 to 1.25 ± 0.65 ng/mL; *P* = .01; Fig. 1E). Changes in BT levels between the second and the baseline determination (Δ BT) were therefore different between PSPA-nt and PA-nt patients (+0.33 ± 0.55 vs −0.26 ± 0.60 ng/mL; *P* = .0088; Fig. 1F).

In PSPA-nt men, FSH and LH levels also significantly increased from the baseline to the time of prolactin normalization (3.6 ± 2.1 to 5.8 ± 3.6 IU/L, *P* = .001; and 2.5 ± 1.2 to 3.6 ± 1.9 IU/L, *P* = .016, respectively; Table 2). By contrast, no changes were observed in the peripheral concentrations of the testicular peptides inhibin B, AMH, and INSL3, as well in those of SHBG (Table 2).

**Table 2.** Longitudinal changes of hormone levels in patients with a prolactin-secreting pituitary adenoma and testosterone levels in the normal range

	Number of pairs	Values at baseline (pairs only)	Values at prolactin normalization (pairs only)	*P* value
Prolactin (ng/mL)	25	1514 ± 2555	10.6 ± 8.1	**<.0001**
Total testosterone (ng/mL)	25	4.6 ± 1.3	5.9 ± 1.8	**.0066**
Bioavailable testosterone (ng/mL)	9	1.29 ± 0.51	1.62 ± 0.64	.2
SHBG (nmol/L)	9	35.9 ± 12.5	44.0 ± 19.4	.14
FSH (IU/L)	18	3.6 ± 2.1	5.8 ± 3.6	**.001**
LH (IU/L)	19	2.5 ± 1.2	3.6 ± 1.9	**.016**

Bold values indicate significant differences.

**Figure 2.**
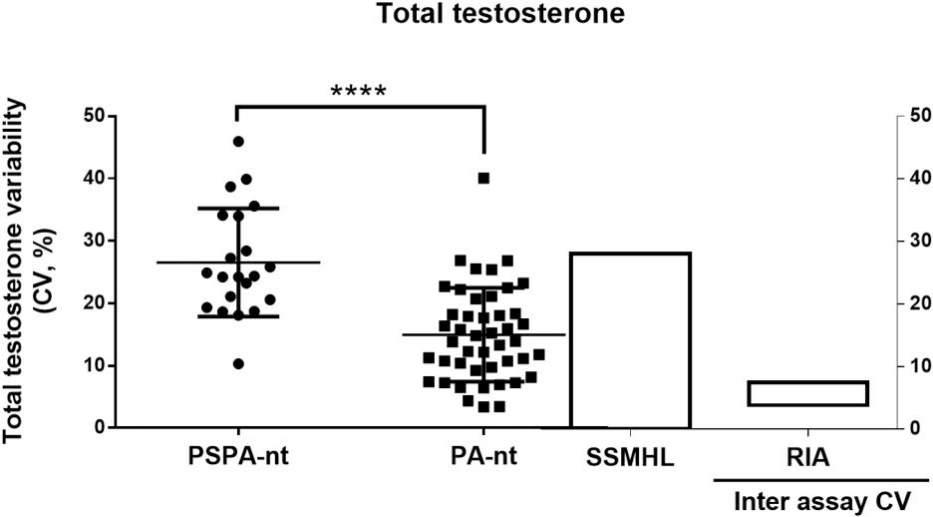
Variability of testosterone levels in PSPA-nt and PA-nt patients having multiple measurements of total testosterone. We included patients having 4 or more total testosterone measurements over the follow-up period. Coefficients of variations (CV, %) were calculated as reported in the Methods section. Individual points of CV for PSPA-nt (including testosterone measurement both before and after normalization of PRL levels, n = 21) and PA-nt (n = 45) patients are reported. The SSMHL bar represents the min-max CV obtained from the steady-state mean hormone levels calculated over 3 repeated measurements in healthy controls (data from Brambilla et al, ref. 27). The inter assay CV for the measurements of total testosterone by means of the used RIA method is provided (box indicating the min-max of the intra assay CV for the low, middle, and high quality control serum). SSMHL, steady-state mean hormone levels. ****P < .0001.

To provide further evidence that the observed testosterone changes did not reflect intrinsic biologic variations, we individualized PSPA-nt (n = 21) and PA-nt (n = 45) patients having 4 or more total testosterone measurements over the follow-up period to calculate the CVs (%). As expected, the CVs of total testosterone levels were higher in PSPA-nt than in PA-nt patients (26.5 ± 8.7% vs 14.9 ± 7.5%; *P* < .0001; Fig. 2). Total testosterone CVs in PA-nt patients were similar to those reported in normal individuals (Fig. 2). In addition, up to 20% of CVs values in PA-nt patients remained within the interassay range (vs none of PSPA-nt; Fig. 2).

### Clinical Signs of Testosterone Deficiency

To understand if the observed hormonal changes in PSPA-nt patients had any clinical relevance, signs and symptoms of TD were collected and graded according to the Endocrine Society guidelines [11]. Overall, 18 (72.0%) PSPA-nt and 24 (39.3%) PA-nt patients complained about at least 1 clinical sign of TD at baseline (*P* = .0086, Supplementary Table S2 [13]). Twelve (48.0%) PSPA-nt and 11 (18.0%) PA-nt individuals described at least 2 symptoms of TD (*P* = .007, Supplementary Table S2 [13]).

**Figure 3.**
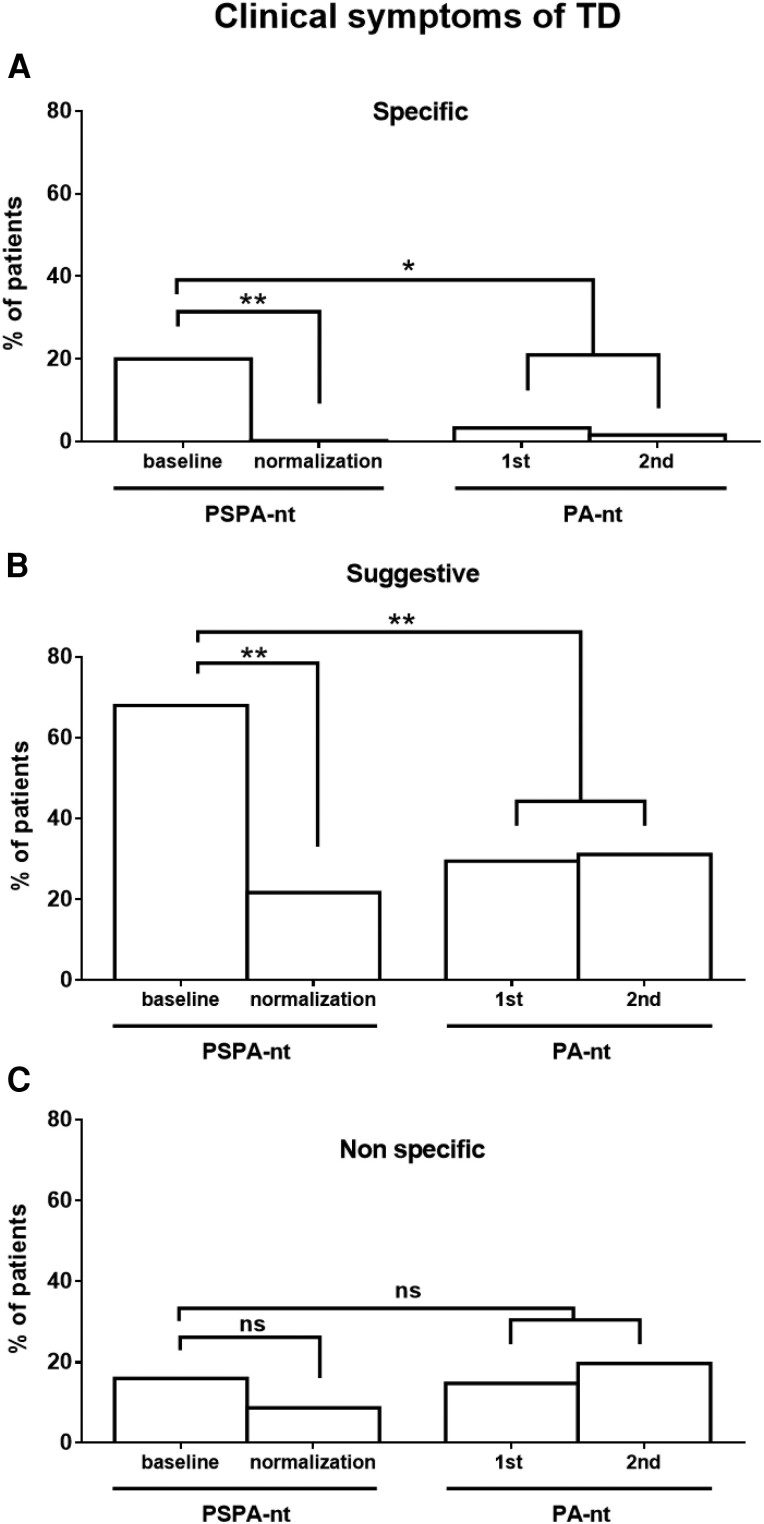
Prevalence and evolution of TD symptoms in patients with PSPA-nt or nonprolactin-secreting pituitary adenoma. Bars represent the percentage of patients complaining of specific (A), suggestive (B), and nonspecific (C) signs and symptoms of testosterone deficiency (see the Methods section and Supplementary Table S2 for more details [13]). *P < .05; **P < .01; ns, not significant.

At baseline, specific signs and symptoms of TD were present in 5 (20.0%) PSPA-nt and in 2 (3.3%) PA-nt patients (*P* = .02 by Fisher test, Fig. 3A and Table 3). In PSPA-nt patients, specific symptoms of TD included loss of body hair and reduced gonadal volume, whereas the 2 PA-nt patients complained about the presence of small testicles (Table 3). The proportion of PSPA-nt patients having specific signs of TD decreased from 28% (at baseline) to 0% at the time of prolactin normalization (*P* = .0096), whereas their rate was unchanged in PA-nt patients (3.3 to 1.6%, Fig. 3A).

**Table 3.** Signs and symptoms of testosterone deficiency in PSPA-nt and PA-nt patients

	PSPA-nt (n = 25)	PA-nt (n = 61)
**Specific, n (%)**	**5 (20.0)** ^***a***^	**2 (3.3)**
Loss of body (axillary and pubic) hair	5	0
Reduced testes volume (<6 mL)	2	2
**Suggestive, n (%)**	**17 (68.0)** ^***b***^	**18 (29.5)**
Reduced sexual desire (libido) and activity	10	12
Decreased spontaneous erections, erectile dysfunction	8	9
Breast discomfort, gynecomastia	7	0
Eunuchoidal body proportions	0	0
Infertility, low sperm count	0	0
Height loss, low-trauma fracture, low BMD	0	0
Hot flushes, sweats	1	7
**Nonspecific, n (%)**	**5 (20.0)**	**9 (14.8)**
Decreased energy, motivation, initiative, and self-confidence	1	0
Feeling sad, depressed mood, persistent low-grade depressive disorder	0	0
Poor concentration and memory	1	0
Sleep disturbance, increased sleepiness	1	1
Mild unexplained anemia (normochromic, normocytic)	1	5
Reduced muscle bulk and strength	0	0
Increased body fat, increased body mass index	2	3

Values showing the prevalence of overall specific, suggestive and non specific symptoms are boldfaced.

Abbreviations: BMD, bone mineral density; PA-nt, patients with a nonprolactin-secreting pituitary neoplasm and testosterone levels in the normal range (see also Patients and Methods section); PSPA-nt, patients with a prolactin-secreting pituitary adenoma and testosterone levels in the normal range.

^*a*^ *P* = .02.

^*b*^ *P* = .0015 (see also text and Fig. 3A,b). Other details on the cumulative prevalence of TD signs are available in Supplementary Table S2 [13].

The proportion of patients manifesting suggestive signs of TD was also higher in PSPA-nt than in PA-nt patients at baseline (68% vs 29.5%, *P* = .0015, Fig. 3B). Most symptoms accounted for impairment in sexual functions or gynecomastia (Table 3). No complaints of infertility or low-trauma fractures were noticed in either group (Table 3). The proportion of patients complaining about suggestive signs of TD decreased from 68% to 21.7% following prolactin normalization in PSPA-nt patients (*P* = .0009, Fig. 3B). By contrast, the prevalence of suggestive signs of TD did not significantly change in PA-nt individuals (from 29.5 to 31.1%, see Fig. 3B).

The proportion of patients declaring nonspecific signs of TD was not different between PSPA-nt and PA-nt patients (16% vs 14.8%; *P* = 1.0; Fig. 3C) and did not significantly change from the baseline to the last clinical visit in the 2 groups (Fig. 3C).

Interestingly, a clinical improvement occurred in 63.2% of patients having a coincident biochemical improvement. By contrast, improvement of TD symptoms was observed in only 33.3% of patients with no testosterone increase (data not shown).

### Predictors of Testosterone Improvement in PSPA-nt Patients

**Figure 4.**
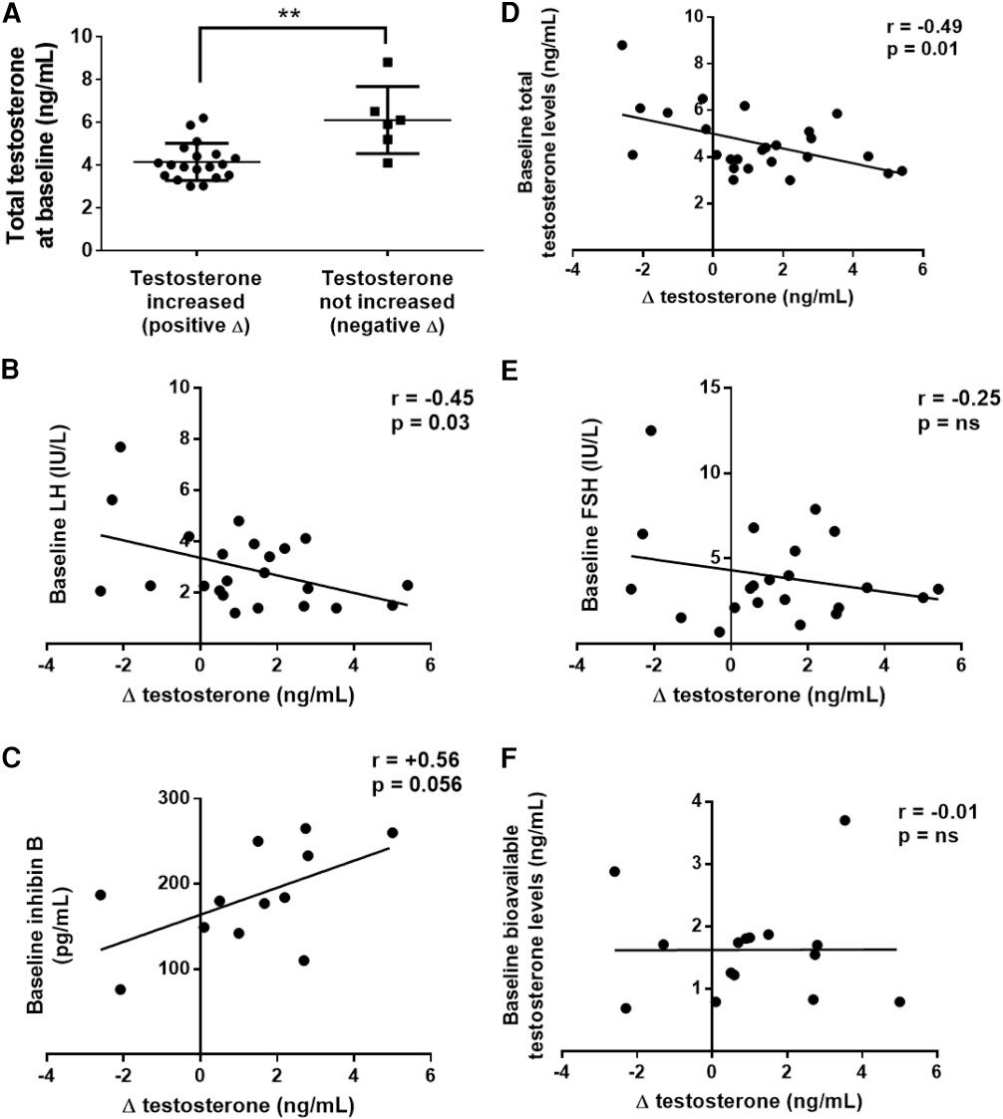
Predictors of testosterone increase in PSPA-nt patients. Total testosterone concentrations at baseline in PSPA-nt patients showing (left) and not showing (right) an increase in testosterone levels at the time of prolactin normalization (A). Correlation between total testosterone changes (Δ testosterone, ng/mL) and baseline total testosterone (B), baseline LH (C), baseline FSH (D), baseline inhibin B (E), and baseline bioavailable testosterone (F) levels. Data are presented as individual points (A), and as linear regressions (panels B-F) with correlation coefficient (*r*) and significance (*P*). **P < .01.

Finally, we attempted to find predictors of testosterone increases in PSPA-nt patients following prolactin normalization. Baseline total testosterone levels were lower in PSPA-nt patients undergoing testosterone increases during the follow-up (positive delta; n = 19; 4.1 ± 0.87 ng/mL) than in those with no improvement (even or negative delta; n = 6; 6.1 ± 1.57 ng/mL; *P* = .002; Fig. 4A). In line with these findings, changes in total testosterone levels (Δ testosterone) negatively correlated with baseline total testosterone levels (*r* = −0.49; *P* = .01; Fig. 4B). Δ testosterone (ng/mL) negatively correlated with baseline LH (n = 25; *r* = −0.45; *P* = .03; Fig. 4C) but not with baseline FSH (n = 25; *r* = −0.25; *P* = .27; Fig. 4D). A trend to a positive correlation was observed between Δ testosterone and baseline inhibin B levels (n = 12; *r* = +0.56; *P* = .056; Fig. 4E). Δ testosterone did not correlate with baseline AMH, INSL3 (n = 12 and n = 4, respectively; data not shown), or baseline BT levels (n = 15, Fig. 4F). Importantly, Δ testosterone did not correlate with baseline prolactin levels (n = 25, *P* = .12). Finally, Δ testosterone levels did not correlate with other clinical parameters, like the age at diagnosis (n = 25, *P* = .79), the BMI (n = 25, *P* = .31), the maximal diameter of the pituitary adenoma (n = 25, *P* = .86), the testicular volume (n = 11, *P* = .84), or the follow-up duration (n = 25, *P* = .94, data not shown).

## Discussion

Prolactin excess is a major cause of acquired hypogonadotropic hypogonadism in men as well as in women [3, 4, 20]. In men, high circulating prolactin levels, like those found in patients harboring a PSPA, are associated with TD, a morbid syndrome characterized by a range of signs and symptoms, and with several long-term clinical consequences [11, 12, 14]. Acquired hypogonadotropic hypogonadism and TD are reported in 62% to 97% of men harboring PSPA, according to different studies [21-25]. In addition, approximately 60% to 70% of hypogonadal patients with PSPA are reported to recover their testosterone levels after a successful (ie, prolactin-normalizing) treatment [8]. Main factors implicated in testosterone recovery in these patients include small tumor size, high baseline testosterone levels, and an early and robust prolactin decrease following the administration of dopaminergic agents [8, 26].

A minority of patients, however, maintain a significant testicular steroidogenic activity, as demonstrated by the presence of total testosterone concentrations in the normal range despite the presence of high prolactin levels. Little information is available about the causes of normal testosterone secretion in these patients and the evolutive changes over time [9, 10]. We thus attempted to understand the testosterone dynamics and the clinical impact of prolactin normalization in patients with PSPA having total testosterone in the normal range. From a large cohort of men with PSPA, we only selected those having testosterone levels in the normal range and assessed the presence of graded signs and symptoms of TD at baseline and following prolactin normalization. To take into account the intraindividual variability of testosterone measurements and the fluctuations attributed to a range of factors as well as incident comorbidities [27], we included a population group harboring other pituitary neoplasms, presenting similar baseline characteristics, and undergoing a similar follow-up.

Following prolactin normalization, we observed a marked increase in testosterone levels in men with PSPA-nt, which was not found in PA-nt. The difference between the last and the first measurement was therefore higher in PSPA-nt than in PA-nt men, whose testosterone longitudinal changes were negligible over the same follow-up duration. Testosterone variability, expressed as CVs over multiple determinations, was more intense in PSPA-nt than in PA-nt individuals. It is true that, although the determination of total testosterone is commonly recommended as a first-line approach to establish hypogonadism in individuals with symptoms of TD, free and bioavailable forms contribute illustrating the androgenic status, especially in individuals with nutritional imbalances or other conditions able to modify transporting proteins, notably the SHBG [28]. Although BT measurements did not increase in the PSPA-nt group, likely because of the small sample size (n = 9 for paired observations), the difference between the last and the first measurement (Δ BT) was higher in PSPA-nt than in PA-nt individuals, similar to that observed for total testosterone. In addition, beyond testosterone, LH, and FSH levels also significantly increased in PSPA-nt patients following prolactin normalization, indicating that the observed testosterone changes truly reflected the recovery of the HPG function.

Importantly, clinical improvements paralleled the observed biochemical modifications. We graded the presence of TD symptoms according to established guidelines [11, 12]. At baseline, the proportion of patients complaining about the presence of specific and suggestive symptoms of TD was clearly higher in PSPA-nt than in PA-nt patients. In addition, contrary to PA-nt patients, the proportion of PSPA-nt experiencing specific and suggestive symptoms of TD patients significantly decreased after a successful treatment aiming at normalizing prolactin.

Our study therefore demonstrates a clinical and biochemical improvement of the HPG axis in patients with PSPA and normal baseline testosterone levels, expanding previous reports including small population samples [9, 10]. Shimon et al reported, in a subgroup of 9 patients with baseline total testosterone levels ≥2.6 ng/mL, increases in total testosterone after treatment [9]. They also reported that sexual function improved in these patients; however, no details on which signs and symptoms of TD were measured [9]. The authors herein used a relatively low threshold to define TD, which included patients commonly defined as hypogonadal at baseline. Ono et al reported 10 eugonadal men with PSPA undergoing testosterone increases following cabergoline treatment. However, they did not report the extent and the clinical impact of these changes because the exploration of TD in these patients was outside the scope of their study [10].

Beyond disorders of libido, sexual activity, and erectile dysfunction, patients with PSPA-nt frequently complained about additional signs of TD, like breast discomfort or gynecomastia and loss of body hair. As expected, the proportion of subjects complaining about nonspecific symptoms, like mood disorders, sleep disturbances or fatigue, was not different between the 2 populations. The overall prevalence of signs and symptoms of TD is estimated at approximately 6% in a general population sample of similar age [29], which is lower than that observed in our 2 groups (both PSPA-nt and PA-nt). It is possible that the medical burden of patients carrying pituitary lesions of any type may in part explain the high prevalence of these symptoms. Interestingly, no patient in our groups consulted for infertility. This probably reflects the lack of fertility concerns in our patients, whose ages broadly exceeded those of paternity in the French general population. Alternatively, it is possible that the occurrence of severe sperm abnormalities requires a more profound suppression of the HPG axis, as is the case for other forms of central hypogonadism [30].

The reasons underpinning the observed testosterone dynamics in PSPA-nt patients are uncertain. We hypothesize that, in healthy individuals, the homeostasis of gonadotropic axis is secured by the presence of relatively steady concentrations of testosterone. Indeed, beyond intradiurnal and seasonal variations, testosterone levels have been shown to remain rather stable on relatively short follow-up durations [31, 32]. Longitudinal evaluation of total testosterone levels in individuals from the Baltimore Longitudinal Study of Aging also showed minimal variations in total testosterone levels in study participants, except in those having additional comorbidities or developing acute or chronic illnesses [27]. Therefore, the upward shift of testosterone levels observed in our patients is biochemically relevant and may reflect a change in their gonadotropic homeostasis. Testosterone levels, albeit in the normal range, should thus be carefully evaluated in patients with high circulating prolactin levels.

The suppression of gonadotropic axis in men with PSPA depends in part on a functional inhibition and in part on the compressive effect of the tumor at the pituitary level [8]. The former action is deemed to pass through a kisspeptin-driven circuit within the infundibular/arcuate region. Dual-label in situ hybridization studies have shown colocalization of prolactin receptor transcripts in subsets of periventricular neurons also expressing kisspeptin [33]. Exogenous kisspeptin administration is able to rescue gonadotropin secretion in humans and in rodents exposed to prolactin excess [6, 34]. The latter event is more likely to occur when the PSPA is sufficiently large to produce a structural damage on the neighboring anterior pituitary. The presence of a concomitant pituitary deficiency (ie, on pituitary axes others than the HPG axis) indirectly supports this hypothesis. Patients with compression of gonadotropic lineage within the pituitary are supposed to less or not recover from hypogonadism despite the normalization of prolactin [8].

Although the rise in total testosterone level is clear on average, its extent is somehow variable, with a subset of individuals with no increases despite a complete normalization of prolactin at the last visit. We thus attempted to find predictors of testosterone improvements in PSPA-nt patients. Baseline total testosterone and baseline LH levels were the strongest predictors of testosterone increases in PSPA-nt patients. The lower the baseline testosterone and LH levels, the higher the testosterone increase at the follow-up. These findings, apparently contrasting with the previous assumption that low testosterone concentrations predict no recovery of the HPG axis [4, 5, 8], strongly indicate a functional HPG disruption in these patients. Indeed, in case of profound TD (associated with very low testosterone and gonadotropin levels), the likelihood of a structural damage of gonadotropes increases, and with that the absence of HPG recovery. In our series, the tumor diameter did not correlate with testosterone increases, indicating that structural damage of gonadotropes in PSPA-nt is trivial, instead validating the hypothesis of a functional hypogonadism. On the other hand, patients having testosterone levels at the upper end are unlikely to observe further increases. The resilience of the HPG axis in these latter patients is intriguing and raises questions on which factor confers protection. Circulating prolactin levels at baseline were not different between patients improving and in those not improving testosterone levels, indicating the absence of a threshold effect and arguing for an inter-individual intrinsic resilience of the HPG axis. More studies are needed to elucidate the mechanisms underlying the HPG axis resistance to high prolactin levels in these patients.

Thresholds to biochemically define TD are numerous, but most studies converge to designate concentrations at 2.7 or 3.0 ng/mL (9.4 and 10.4 nmol/L, respectively) as those bearing the maximal sensitivity to predict the occurrence of signs and symptoms of TD [29, 35, 36]. We are thus dealing with a specific population, with a high prevalence of TD symptoms despite having total testosterone concentrations >3.0 ng/mL. For testosterone measurements, we did not use mass spectrometry (gas chromatography/tandem mass spectrometry or liquid chromatography/tandem mass spectrometry), which is considered the most accurate. However, robust immunoassays are nowadays reported to be also highly reliable and provide good accuracy, especially when dealing with values within the normal range, as those reported here [37].

The main strength of our study lies on the methodology. All patients were sampled at the same referral center, and interviews on TD symptoms in PSPA-nt and PA-nt patients were similarly structured. In addition, we chose to include an age, BMI, and follow-up duration matched population to control either testosterone or clinical symptoms longitudinal changes. In addition, because the threshold by which prolactin inhibits the HPG axis is uncertain, we decided to exclude PA-nt patients having mild prolactin elevation, believed to be associated with a pituitary stalk compression [38]. For the same reason, we included only PSPA patients having stringently normalized their prolactin levels (<20 ng/mL) at the last visit. The main limitation of our study lies in the relatively small sample of patients. Multicentric studies including more individuals should be useful to validate our findings. Per the retrospective nature of our study, a number of biological pairs were missing. Importantly, we did not assess estradiol levels in our patients. Yet, estrogen deficiency, which is part of hypogonadotropic hypogonadism in men, is considered responsible for some of the clinical features seen in hypogonadal patients, such as impairment in body composition and sexual dysfunctions [35, 39]. Finally, apart from biochemical analyses and clinical symptoms over short follow-up periods, our study was not designed to deal with long-term consequences of androgen deficiency, like disorders of bone mineral content, muscle strength, and performance or cardiometabolic risks.

Our findings, however, have noteworthy implications in common practice. When dealing with patients with PSPA deemed to be eugonadal, we advise to seek for the presence of additional symptoms of TD. The presence of specific or suggestive symptoms of TD should not be minimized or overlooked, especially in patients having baseline total testosterone at the lower end (typically around 4 ng/mL or 13.9 nmol/L). Patients should be advised that these symptoms may attenuate or disappear following an effective prolactin-lowering treatment. Experienced physicians dealing with large PSPA also know that tumor control takes priority over biochemical normalization, and, in numerous cases, prolactin levels never completely normalize despite extensive tumor shrinkage [3, 40, 41]. Sometimes, supra normal prolactin levels associated with persistent tumor mass are even desired, in an attempt to prevent a breach at the level of the sellar floor, fragilized by the tumor [42]. Androgen substitution should therefore be considered and offered in these patients to tackle and relieve TD symptoms, to improve their overall quality of life, and potentially prevent long-term consequences of sex steroid deficiency.

## Data Availability

Data generated or analyzed during the study are available from the corresponding author by request.

## Abbreviations

BMI: body mass index
BT: bioavailable testosterone
CV: coefficient of variation
HPG: hypothalamus-pituitary-gonadal
PA-nt: pituitary neoplasms and normal testosterone levels
PSPA: prolactin-secreting pituitary adenoma
PSPA-nt: men with PSPA and repeatedly normal testosterone levels at baseline
TD: testosterone deficiency
